# From the other perspective: Behavioural factors associated with UK sheep farmers’ attitudes towards antibiotic use and antibiotic resistance

**DOI:** 10.1371/journal.pone.0251439

**Published:** 2021-05-27

**Authors:** Charlotte Doidge, Eliana Lima, Fiona Lovatt, Chris Hudson, Jasmeet Kaler

**Affiliations:** School of Veterinary Medicine and Science, University of Nottingham, Sutton Bonington, United Kingdom; University of Lincoln, UNITED KINGDOM

## Abstract

Research suggests that many sheep farmers continue to carry out traditional antibiotic use practices despite new ’good practice’ recommendations. The aim of this study was to group farmers depending on their attitudes around antibiotic use and antibiotic resistance, and determine the behaviours that are associated with the farmers in these groups. In 2017, a flock health survey was sent to British sheep farmers. K-means cluster analysis was used to identify groups of farmers with similar attitudes towards antibiotic use and resistance. A multivariable logistic regression model was built to determine the associations between farmers’ past behaviours and their antibiotic attitude group. There were 461 responses. Two groups of farmers were identified based on their antibiotic attitudes. Cluster 1 were defined as the "discordant" group who had positive views of using antibiotics prophylactically and negative views of reducing antibiotic use. Cluster 2 were defined as the "concordant" group who were positive about reducing antibiotic use and had negative views about using antibiotics prophylactically. Using antibiotics in all lambs (OR = 2.689, CI = 1.571, 4.603), using antibiotics in all ewes (OR = 3.388, CI = 1.318, 8.706), always trimming diseased feet over the past three years (OR = 2.487, CI = 1.459, 4.238), not using a computer to record information over the past three years (OR = 1.996, CI = 1.179, 3.381), not changing worming practices over the past three years (OR = 1.879, CI = 1.144, 3.087), and farmers’ perceptions that their sheep flock did not make a financial loss in the past three years (OR = 2.088, CI = 1.079, 4.040) were significantly associated with belonging to the discordant group. Talking to their veterinarian about antibiotic use or the frequency of veterinary visits were not associated with antibiotic attitude group. These results suggest that farmers who had attitudes relating to antibiotic use that did not align with current recommendations carried out more traditional practices, which were strengthened by their positive perceptions of profitability.

## Introduction

In light of the global antibiotic resistance crisis, measures are being taken to ensure the prudent use of antibiotics, with a particular political interest in reducing avoidable use in agriculture [1].There are over 70,000 breeding ewe holdings in the UK making up around 40% of the total livestock biomass in the UK [2,3]. Therefore, although the sheep sector is thought to be a low use sector based on limited data available [4], sheep production plays a significant role in the overall agricultural antibiotic use figures for the UK. The latest figures from the Veterinary Medicines Directorate indicated that sale of antibiotic for use across all food producing species in the UK was 31.0mg/kg in 2019 [5].

Quantification of antibiotic use in the sheep sector is challenging as sheep producers often have additional enterprises such as beef production, which makes it difficult to distinguish what species a drug has been used in. Additionally, until recently, there was no central location for sheep antibiotic usage data to be collated in the UK. The Royal College of Veterinary Surgeons legislation states that antibiotics on UK farms must be prescribed by a veterinarian who has the animals ‘under their care’ [6]. Many sheep farms are part of farm assurance schemes which means that they must adhere to additional requirements such as an annual flock health review, where their antibiotic use data is reviewed by their veterinarian [7]. At present there is no set way of record keeping and farmers may choose to record using a paper based medicine book or computer based using a spreadsheet or computer programme. The Agriculture and Horticulture Development Board (AHDB) has recently launched a medicine hub which aims to collate national medicine use for the dairy, beef and sheep sectors [8]. A target has been set to capture antibiotic use data from 10% of UK sheep flocks by 2024 [4].

Whilst antibiotic use is thought to be low on sheep farms [4,9], there are still opportunities for improved antibiotic stewardship. Antibiotics may be used to prevent disease (prophylaxis, e.g., to prevent abortion in ewes or neonatal diseases in lambs), to control disease using group treatments in an outbreak situation (metaphylaxis, e.g., to control an outbreak of pneumonia), or to treat individual sick animals. The majority of antibiotic use within the sheep sector is for the treatment of lameness [4]. The five-point plan to control and reduce lameness within flocks was introduced in 2014 and is widely recommended to farmers by the sheep industry [10]. The plan advises practices including culling persistently lame sheep, vaccinating for footrot, prompt treatment with antibiotics and not trimming feet. These practices have been shown to reduce the lameness prevalence within flocks [11–14]. However, the uptake of the five-point plan by farmers is very low [14,15]. This may be because foot trimming was traditionally best practice that has been embedded as a cultural norm of what a ’good farmer’ does, which makes the behaviour resistant to change [16,17].

As highlighted in The Responsible Use of Medicines in Agriculture Alliance (RUMA) reports, one of the "hotspots" where antibiotic use could be reduced in the sheep sector is neonatal lamb diseases, such as watery mouth (colibacillosis) and joint ill (suppurative polyarthritis) [4]. Historically, the prophylactic (preventative) use of antibiotics in neonatal lambs to control watery mouth and joint ill was common practice but this is now actively discouraged [4]. In 2016, 31% of UK sheep farms surveyed were still using antibiotics for prevention of disease in neonatal lambs [18].

The routine use of antibiotics in pregnant ewes to prevent abortion is also considered to be a ‘hotspot’ for unnecessary use [4]. As vaccines are available for the most common causative agents, *Chlamydia abortus* and *Toxoplasma gondii*, antibiotic use for the control of abortion is often deemed unnecessary [19]. Management practices such as keeping a closed flock, buying from accredited sources and isolating recently aborted ewes can also help to prevent the spread of abortion. Results from a 2007 survey suggested that 9% of sheep farmers in the UK used antibiotics to control abortion [20]. The characteristics of farms that use antibiotics for prevention of either abortion or neonatal diseases is unknown and needs to be investigated further to understand why farmers continue to use antibiotics prophylactically.

Most research into farmer antibiotic use behaviour focusses on the way farmers’ attitudes influence their behaviour [21,22]. This is because many social-psychological theories, including perhaps the most utilised theory—the Theory of Planned Behaviour—propose that factors such as attitudes and perceptions influence behaviour [23]. One limitation of previous studies is that they only investigate the association between attitudes and a single behaviour. Focusing on a single behaviour may lead to biased assessments because behaviours often have several interdependencies [24]. One method of assessing the associations of multiple attitudes and behaviours at the same time is to cluster those with similarly held attitudes into groups and then assess the profile of these groups in terms of behavioural and structural characteristics [25,26].

There is evidence that the association between attitude and behaviour is bidirectional and that behaviours can influence attitudes. This is particularly the case for undesirable behaviours such as smoking [27] and binge drinking [28]. Thus, past behaviour is one of the origins of attitude formation [29]. Furthermore, there is evidence that behaviour can influence attitudes more strongly than the reverse [30]. Social science research into farmers’ antibiotic use focusses on the influence of farmer attitudes on their antibiotic use behaviour [21,22,31]. However, traditional embedded practices such as prophylactic antibiotic use and foot trimming are repetitive, habitual behaviours which occur year after year, and farmers will learn and adapt from their previous experiences. Previous studies around farmers’ attitudes to antibiotics have not explored the possible associations with past behaviour.

In this introduction we have considered multiple antibiotic practices that farmers continue to carry out despite new ’good practice’ recommendations. The aim of this study was to group farmers depending on their attitudes around antibiotic use and antibiotic resistance and then determine the behaviours that are associated with the farmers in these groups.

## Methods

### Survey design

The survey was developed as part of a larger project and was designed by EL, JK and FL and the survey design has previously been described in detail [32]. This paper investigated a subsection of data on antibiotic use which has not yet been explored. Therefore, this paper will only refer to the sections used to explore associations with antibiotic use attitudes. The survey included sections on farm characteristics, farmer demographics, flock health management, antibiotic usage practices, opinions on antibiotic use and antibiotic resistance. The respondents were asked to answer the questions relating to practices implemented between September 2016 and August 2017. Below is an overview of each section of the survey used to explore associations with antibiotic attitudes.

#### Farmer and farm characteristics

This section included questions on the farmer’s characteristics such as age, number of years farming sheep and level of education. The farm characteristics included number of mature ewes, production system type and size of farm. Farmers were asked whether their sheep flock had made a profit, loss or broken even over the past three years. They were also asked if they had changed any farm practices over the past three years.

#### Health and performance of the flock

Respondents were asked about their management practices related to lameness control. This included vaccination against footrot, time from observation to treatment of lame sheep, foot trimming, use of antibiotic injection, use of foot spray and separation of lame sheep.

Other questions related to health management and performance were worming of lambs and ewes, culling practices, and data recording practices. Frequency of advice from a veterinarian was included.

#### Antibiotic use practices

Farmers were asked about the proportion of lambs they treated with antibiotics for the treatment and prevention of watery mouth, joint ill and cases of ill lambs (e.g. pneumonia) in the lambing season between September 2016 and August 2017. They were asked about the type of antibiotic given which included oxytetracycline injectable, white injectable (e.g. penicillins), other injectable, oral antibiotics and antibiotic pills. Photographs and manufacturer names of antibiotics were included in this section for clarification. Farmers were also asked about the proportion of ewes they treated with antibiotics for the treatment or prevention of abortion, problems around lambing, lameness, illness (e.g. pneumonia) and mastitis.

#### Opinions on antibiotic use

Respondents were asked to rate a series of sixteen statements around attitudes toward antibiotic use and resistance, from 1 = Strongly disagree to 5 = Strongly agree. A don’t know option was also available to select. The statements were informed from the wider literature on attitudes towards antibiotics [21,33,34].

### Distribution of survey

The distribution of this survey has been previously described in [32]. The target population for the sheep survey were farms in the UK who supplied lambs to a British retailer through two abattoirs (n = 830). The target farms were sent links to the online survey by the two participating abattoirs in November 2017. A reminder was sent to non-responders in early January 2018 before the survey closed at the end of January 2018. Farmers were informed that the anonymised data generated from this survey were to be used and published for research purposes. Participation was voluntary and informed consent was gathered at the beginning of the survey by farmers agreeing to continue with the survey.

The study was approved by the University of Nottingham School of Veterinary Medicine and Science Ethics Committee (no 1850 160916).

### Data analysis

Data cleaning and data analysis including descriptive statistics, cluster analysis and logistic regression were carried out in Stata 16 (Stata SE/16.1, Stata Corp., College Station, TX, USA). Where there were duplicate entries from the same farm, the most complete response was kept for analysis. General descriptive results from this survey have previously been published [32]. However, due to differences in the data cleaning and analysis requirements, this paper uses a larger number of respondents. Therefore, the descriptive results are reported again in case of differences in the sample population.

#### Cluster analysis

Cluster analysis was performed to identify groups of farmers with similar attitudes towards antibiotic use and resistance. The farmers were grouped based on their ratings of the sixteen statements relating to antibiotic use and resistance, from 1 (strongly disagree) to 5 (strongly agree). A small proportion (0.7–5.4%) of "don’t know" responses were reported for nine statements. Any “don’t know” responses were recoded as 3 (neutral) for cluster analysis. Cluster analysis was also run with the "don’t know" responses removed and there was no significant differences in the results. Therefore, the set of responses with "don’t know" coded as neutral were used as less respondents were removed from the analysis. Four respondents were removed at this stage because of missing ratings for the statements.

K-means clustering uses an iterative algorithm to partition *n* observations into *k* clusters. A limitation of k-means cluster analysis is that the number of clusters must be defined *a priori* [35]. Therefore, hierarchical cluster analysis using Ward’s method was carried out initially. A dendrogram was produced to estimate the optimum number of clusters [36]. This estimate was then used as the prespecified number of clusters in K-means cluster analysis. The Euclidian distance measure was used to determine the dissimilarity between observations. Sensitivity analyses was carried out using different clustering solutions to ensure the appropriate number of clusters was determined. The optimum number of clusters was two, as this achieved distinct clusters with similar group sizes and were easily interpretable upon inspection. The clusters were labelled based on their attitude alignment with current antibiotic use recommendations.

#### Multivariable logistic regression

The dependent variable of interest for the multivariable logistic regression was the antibiotic attitude cluster group, where 1 was the "discordant group" whose attitudes did not align with current antibiotic use recommendations (e.g. thinking that using antibiotics for prevention was acceptable) and 0 was the "concordant group" whose attitudes did align well with current antibiotic recommendations. Potential independent variables were those relating to past and current management practices. New variables were created for past practices by inspecting farmers’ responses relating to changes in management practices. If farmers selected that they had not changed any practices over the past three years, then it was assumed that the practices stated by farmers in the survey were carried out for the past three years. A univariable analysis was carried out and variables with p≤0.1 were considered for the multivariable analysis. A forward selection stepwise model building approach was used, where only variables with p≤0.05 were selected to remain in the model. The multivariable logistic regression model took the form of:

$${cluster\;group}_{i}∼Bernoulli(mean=\mu_{j})$$

$$\ln\left(\frac{\mu_{i}}{1-\mu_{i}}\right)=\alpha +\beta_{i}x_{i}$$

Where *cluster group_i_* is the antibiotic attitude cluster group the ith farmer belongs to, *μ_i_* is the fitted probability of the outcome, α is the intercept, ***β_i_*** is the vector of coefficients corresponding to the vector of predictor variables ***x_i_***.

The Hosmer-Lemeshow test was carried out to test model fit. The Variance Inflation Factor (VIF) and the tolerance were inspected for collinearity between variables.

## Results

A total of 461 usable responses were received, resulting in a 55.5% useable response rate. Of the respondents, 43.7% (200/461) were aged between 36 and 55, 40.8% (187/461) were aged over 55 and the remaining 15.5% were under 35 years old (71/461). Most farmers housed their sheep at lambing time (71.6%, 330/461).

The median flock size of farms was 520 (IQR 300–830), and the median grassland area used for sheep was 258 acres (IQR 150–480). Three quarters of sheep farmers also kept beef cattle (75.5%, 248/461). The median number of breeding female beef cows kept was 18 (IQR 0–50) and the median number of other cattle kept was 40 (IQR 0–90). The characteristics of the farm and farmer are presented in Table 1.

**Table 1:** Characteristics of the respondents and their farms.

Characteristic	Percent (N)		
**Age of farmer (years)**			
Under 35	15.5% (71)		
36–55	43.7% (200)		
Over 55	40.8% (187)		
**Highest qualification**			
Secondary school	39.9% (184)		
Technical college	43.2% (199)		
University	16.9% (78)		
**Flock type**^**a**^			
Lowland	36.9% (170)		
Upland	53.8% (248)		
Hill	28.6% (132)		
**Inside lambing**			
Yes	71.6% (330)		
No	28.4% (131)		
	**Median**	**25% IQR**	**75% IQR**
**Number of mature ewes**	520	300	830
**Grassland area used for sheep farming (acres)**	258	150	480
**% time spent working with sheep**	50	35	70
**Number of full time staff**	1	0	2
**Number of mature cows (beef)**	18	0	50
**Number of other cattle (not including dairy)**	40	0	90

^a^ Respondents could select more than one choice.

### Flock productivity

Over the past three years, 11.7% (54/461) of farmers thought that their sheep flock made a financial loss, 33.4% (154/461) thought they were at breakeven point and 45.3% (209/461) thought that they made a profit.

### Changes in management practices

Over the past three years, 53.4% (246/461) of farmers had not changed any management practices on their farm, 21.3% (98/461) had made changes to their culling practices, 18.7% (86/461) had made changes to their recording practices, 26.3% (121/461) had made changes to their worming practices, and 17.1% (79/461) had made other changes to their management practices.

To investigate the changes in worming practices further, chi-squared tests were conducted to identify differences in worming practices between those who changed their practices and those who did not. A chi-squared test indicated that the proportion of farmers using faecal egg counting to judge whether lambs needed worming was significantly different for farmers who had changed their worming practices compared to those that had not (χ^2^(1, N  =  457)  =  66.175, p<0.001).

### Flock advice

In the period September 2016 to August 2017, 6.5% (30/461) of farmers never sought advice about their flock from a veterinarian, 29.3% (135/461) sought advice just once, and 64.2% sought advice at least quarterly (296/461).

### Antibiotic use

Just over half of farmers reported that they had talked about antibiotic usage with their veterinarian between September 2016 and August 2017 (51.4%, 237/461), and just over a quarter that they had talked about antibiotic resistance with their veterinarian (26.7%, 123/461). Many farmers reported that they had not talked about either antibiotic use or resistance with their veterinarian (41.9%, 193/461).

Only 2% (9/461) of farmers thought their antibiotic use was higher than usual between September 2016 and August 2017. Most farmers thought their antibiotic usage was roughly the same as usual (68.8%, 317/461), and the remaining 29.3% (135/461) of farmers thought their antibiotic usage was lower than usual.

Around half of farmers thought their antibiotic use was less than other sheep farms (52.5%, 242/461). Most of the remaining farmers thought that their antibiotic use was approximately the same as other sheep farms (46.9%, 216/461) and three farms thought their antibiotic use was higher than other farms.

Twenty-two percent (102/461) of farmers used antibiotics for prevention of watery mouth, joint ill or illness in all of their lambs, whereas 8.5% (39/461) of farmers used antibiotics for prevention of disease in all ewes.

### Antibiotic attitude clusters

Respondents were clustered into two groups related to the rating of seventeen statements around antibiotic use and antibiotic resistance. The means of how each group rated the statements are presented in Table 2, where 1 represents “strongly disagree” and 5 represents “strongly agree”. There were particularly large differences in the mean ratings between the two clusters for statements relating to the use of antibiotic for prevention of disease and the practicality of alternatives to the use of antibiotics. The group of farmers in antibiotic attitude cluster 1 tended to think that use of antibiotics for prevention was acceptable and that use of antibiotics was the only option for them, as alternatives were difficult or not feasible. Therefore, this cluster was named the "discordant group". Farmers in antibiotic attitude cluster 2 were more optimistic about alternatives to antibiotic use and disagreed with using antibiotics for prevention. This cluster was named the "concordant group".

**Table 2:** Mean ratings of antibiotic statements for two clusters of farmers (1 = strongly disagree, 5 = strongly agree).

	Antibiotic attitude cluster 1 "Discordant group" Mean (SE)	Antibiotic attitude cluster 2 "Concordant group" Mean (SE)
The use of antibiotics is beneficial to prevent disease in my flock	3.811 (0.059)	2.162 (0.068)
The use of antibiotics is beneficial to ensure productivity of my flock	3.712 (0.057)	2.776 (0.080)
The use of antibiotics is beneficial to the welfare of my flock	4.223 (0.039)	3.868 (0.065)
In some flocks, dosing new born lambs with an oral antibiotic is the only way to be sure watery mouth is prevented	3.674 (0.063)	2.785 (0.083)
It is ok to use antibiotics to treat sick animals	4.450 (0.038)	4.518 (0.043)
It is ok to use antibiotics to prevent disease in animals	3.772 (0.057)	2.254 (0.068)
Society thinks farmers use too many antibiotics	3.408 (0.058)	3.842 (0.054)
The media provide a negative view of farmers regarding the use of antibiotics	3.918 (0.053)	4.026 (0.055)
Using less antibiotics makes me a good farmer	3.077 (0.065)	3.535 (0.065)
Preventative use of antibiotics can contribute to antibiotic resistance in sheep	3.704 (0.054)	4.329 (0.049)
Reductions in the use of antibiotics could be achieved through better management	3.584 (0.056)	4.228 (0.051)
Alternatives to antibiotics are not practical to implement	3.202 (0.054)	2.399 (0.059)
People I respect in the industry would approve of reducing the use of antibiotics in my flock over the next year	3.292 (0.053)	3.518 (0.054)
Reducing antibiotic usage in my flock would have costs	3.622 (0.049)	2.899 (0.058)
Curative use of antibiotics can contribute to antibiotic resistance in sheep	3.155 (0.057)	2.785 (0.066)
If every sheep farmer followed best practice, there would be less bacteria resistance to antibiotics in the human population	3.305 (0.054)	3.241 (0.067)
I have the skills and knowledge needed to reduce antibiotic use in my flock in the near future	3.421 (0.045)	3.588 (0.047)
N	226	231

### Multivariable logistic regression model

A multivariable logistic regression model was built to estimate the associations of farmer practices and behaviours on belonging to the discordant antibiotic attitude group. The results are reported in Table 3.

**Table 3:** Results of multivariable logistic regression for the associations of farmer practices and behaviours on farmers belonging to the discordant antibiotic attitude group compared to the concordant attitude group (N = 457).

	N	Odds Ratio (95% CI)	P>z
**Using a computer to record information for past three years**			
Yes	88	Ref	
No	369	1.996 (1.179, 3.381)	0.010
**Did not change worming practices in past three years**			
No	120	Ref	
Yes	337	1.879 (1.144, 3.087)	0.013
**Used antibiotics for prevention of disease in all lambs born on farm in previous year**			
No	356	Ref	
Yes	101	2.689 (1.571, 4.603)	<0.001
**Used antibiotics for prevention of disease in all ewes in previous year**			
No	418	Ref	
Yes	39	3.388 (1.318, 8.706)	0.011
**Always trimmed diseased feet in past three years**			
No	358	Ref	
Yes	99	2.487 (1.459, 4.238)	0.001
**Farmer reported flock made financial loss in past three years**			
Yes	54	Ref	
No	403	2.088 (1.079, 4.040)	0.029
**My antibiotic use was**			
Less than other farms	239	Ref	
Same as other farms	218	2.495 (1.644, 3.785)	<0.001
Talked to veterinarian about antibiotic use or antibiotic resistance in past year*			
Yes	267	Ref	
No	190	0.839 (0.550, 1.281)	0.416
Frequency of veterinarian advice*			
Never	30	Ref	
Only once	133	1.016 (0.419, 2.446)	0.972
At least quarterly	294	0.988 (0.422, 2.310)	0.977
Intercept		0.082 (0.033, 0.205)	<0.001

*Variables used interchangeably in model 1 and model 2.

The odds of belonging to the discordant group doubled when farmers did not use a computer to record information compared with farmers who used a computer for the past three years (CI = 1.179, 3.381), when farmers had not changed their worming practices in the past three years compared to farmers who had changed their worming practices (CI = 1.144, 3.087) and when farmers reported that their sheep flock did not make a financial loss in the past three years (CI = 1.079, 4.040).

Farmers who used antibiotics for prevention of disease in all lambs born on their farm in the previous year had 2.689 times higher odds of belonging to the discordant group compared to farmers who did not use antibiotics in all their lambs (CI = 1.571, 4.603). Farmers who used antibiotics for prevention of disease in all ewes on their farm in the previous year had 3.388 times higher odds of belonging to the discordant group compared to farmers who did not use antibiotic in all their ewes (CI = 1.318, 8.706). Farmers who always trimmed diseased feet for the past three years had 2.487 times higher odds of belonging to the discordant group compared to farmers who did not always trim diseased feet (CI = 1.459, 4.238). Farmers who thought that they used the same amount of antibiotics as other sheep flocks had 2.495 times higher odds of belonging to the discordant group compared with farmers who thought that they used less antibiotics than other sheep flocks (CI = 1.644, 3.785).

The Hosmer-Lemeshow test gave a p-value of 0.5, indicating that the model fit the data well. The VIF and tolerance values of the variables used in the logistic regression indicated that there were no collinearity problems.

A second model included the same variables with the same positive or negative associations as model presented in Table 3. However, "Talked to veterinarian about antibiotics use or antibiotic resistance" was removed from the model because it was not significant. "Frequency of veterinarian advice" was included to investigate whether this had any association with farmers’ attitudes to antibiotics. Reporting that they received veterinarian advice just once (CI = 0.419, 2.446), or at least quarterly (CI = 0.422, 2.310) between the period September 2016 and August 2017 had no significant difference in the odds of a farmer belonging to the discordant group, compared to reporting that they did not receive veterinarian advice in that time period.

## Discussion

This is the first study to investigate how farmers’ past behaviours may be associated with their attitudes towards antibiotic use. The results suggest that farmers could be clustered into two different groups based on their antibiotic attitudes. The "discordant" group were more likely to have negative views of reducing antibiotic use and positive views of using antibiotics for prevention, and therefore have attitudes that are discordant with current antibiotic use recommendations. The "concordant" group were more positive about reducing their antibiotic use and had negative views about using antibiotics for prevention. Belonging to the discordant group was associated with carrying out traditional practices, whereas belonging to the concordant group was associated with carrying out modern practices and recently changing their practices.

The existence of the discordant group of farmers may be explained using the theory of cognitive dissonance within the free choice paradigm, where a decision is freely made by an individual [37]. Once a farmer had chosen to adopt a management practice, such as prophylactic use of antibiotics in lambs, the alternative is no longer available and they are now committed to the chosen practice [38]. They may reject cognitions that are dissonant with the practice such as "Preventative use of antibiotics can contribute to antibiotic resistance in sheep". They may also exaggerate the positives of their chosen practice such as "Use of antibiotics is beneficial to prevent disease in my flock" and downplay the positives of the alternative practices by agreeing to statements such as "Alternatives to antibiotics are not practical to implement". This may explain why farmers who used antibiotics for prevention of disease were more likely to belong to the discordant group. The results show that in 2017 over a fifth of farmers in this study were still using prophylactic antibiotics in all lambs as routine. This is despite the many information sources available to farmers that show that routine prophylactic antibiotic use is rarely appropriate and alternative strategies exists [39]. A similar cognitive dissonance situation has been identified in the control of lameness in sheep, where farmers identified time and cost as barriers towards adopting the recommended control practices, even though such recommendations were actually time- and money-saving [17].

Other traditional practices, such as trimming sheep feet and not changing anthelmintic use, were also associated with belonging to the discordant group. Trimming sheep feet to treat and control lameness is an age-old practice that has been passed down through the generations of farmers [17]. However, this is no longer a recommended practice as it has been shown to increase the lameness prevalence in flocks [11,13]. Similarly, routine anthelmintic use has been used by farmers for a long time and has become part of their everyday practices [40]. Guidelines on sustainable parasite control avoiding non-targeted routine anthelmintic treatments have been developed and promoted [41], and evidence shows that sustainable parasite management was associated with lower anthelmintic use without a lower productivity or higher worm burden [42]. Yet, it is known that farmers may reject this information in favour of their traditional "family farm" identities and perceptions of productivity [16]. Farmers may discard information on recommended practices for parasite or lameness control due to cognitive dissonance, as the information does not align with their traditional practices.

Over half of farmers had not changed any of their farm practices in three years, suggesting that their farm practices may have become habitual over the years. When a behaviour is repeated in a consistent context, automaticity increases [43]. For example, upon the cue of seeing a lame sheep a farmer may be automatically triggered to carry out foot trimming or on the cue of a certain date a farmer may be triggered to treat their sheep with anthelmintic. When a habit is triggered then alternative practices become less cognitively accessible [44]. People will be less open to new information and less likely to seek new information regarding the behaviour or alternatives to the behaviour [45]. This makes changes to recommendations for a new alternative behaviour less noticeable, such as using injectable antibiotics to treat lameness, or determining the need to worm lambs using faecal egg counting. Therefore, habitual behaviour is less susceptible to change. This possible habit formation may be why farmers carrying out traditional practices have attitudes related to antibiotic use that are discordant from current antibiotic use recommendations. According to self-perception theory, people who carry out a habitual behaviour use this behaviour to form their attitudes [46]. For example, a farmer may think "I have always used antibiotics in new born lambs and therefore it must be beneficial". Rejecting dissonant information is easier than breaking a strong habit and so the traditional practices will persist along with their cognitions [45].

The farmers who did not use a computer to record farm information for the past three years were more likely to belong to the discordant group. Electronic recording is a relatively modern practice in sheep farming; for example, electronic identification of sheep became mandatory in the EU in 2010 [47]. Use of electronic recording is more reliable than paper based recording as it reduces the occurrence of mistakes and can make data entry easier [48]. However, many farmers in this study did not use a computer to record information. Kaler and Ruston [49] showed that barriers to technology adoption by sheep farmers included the belief that technology could not replace the skill of a good stockperson and that farmers needed to have a hands-on approach. Thus, farmers who resist the adoption of technology often have traditional views of good farming stock keeping skills [50,51]. Farmers may infer their identity as a farmer based on their past behaviours [46]. They might inspect their past behaviour and deduce that they are the type of farmer that behaves this way [52,53]. Then, this behaviour becomes incorporated into their self-identity.

Farmers who perceived that they did not make a loss from their sheep flock in the past three years were more likely to belong to the discordant group. Although on average sheep and beef farmers typically make smaller profits than other farming businesses, productivity is an important goal of any farm business [54]. According to Bourdieu’s theory of capital, productivity is a sign of a farmer’s economic and cultural capital (i.e. material assets, skills) which is incorporated into their identity as a "good farmer" [55,56]. As their previous practices have been perceived as profitable, these are then linked to the good farming ideal. The cognitions relating to the practices involved, such as antibiotics are beneficial to productivity, may be reinforced because of the positive outcome. This means that they may be less likely to change the cognitions relating to antibiotic use.

Farmers who believed that their antibiotic use was the same as other farms were more likely to belong to the discordant group. Therefore, farmers in the discordant group may think that the way they perceive antibiotic use is similar to other farmers (i.e. a descriptive norm). This is similar to findings in a study of dairy farmers, where perceived norms around mastitis treatment hindered appropriate antibiotic use [33]. Farmers may also perceive that using antibiotics is part of the injunctive norm of being a "good farmer" as they believed antibiotics improve productivity and prevent disease. It is difficult to change behaviour or attitudes if farmers believe they would violate the norms around antibiotic use. However, our study suggests that beliefs that are discordant with current antibiotic use recommendations are not actually the norm amongst sheep farmers. Many farmers belonged to the concordant group whose beliefs aligned with current antibiotic use recommendations. This could help to dismantle the perceived norms within the discordant group [57]. The launch of the new Medicines Hub in the UK to collate antibiotic use in the sheep sector may also help to dismantle social norms around antibiotic use, as farmers will eventually be able to identify how their antibiotic use compares to others [8].

The frequency of veterinarian advice and whether the farmer reported that they had specifically spoken with their veterinarian about antibiotic use were not significantly associated with farmers’ antibiotic attitude group. This may be because veterinarians were not obviously speaking with farmers about appropriate antibiotic use at the time of the study. Studies from pig, poultry and dairy sectors found that veterinarians were a key source of information on antibiotic use for farmers [21,34,58]. This difference between sheep farmers and other farmers might be because some sheep farmers may find their veterinarian’s advice confusing or that it conflicts with other advice [9]. Additionally, some sheep veterinarians may think that it is still acceptable to prescribe routine antibiotics for the prevention of neonatal lamb disease and this view could then be disseminated to their clients [59]. One method of reducing dissonance is through socialising with those that hold cognitions similar to those one wishes to maintain [60]. Therefore, farmers may choose to use a veterinarian based on their views on antibiotics. On the other hand, some farmers may ignore their veterinarian’s advice if it does not align with their practices. We have previously shown that some farmers did not take their veterinarians advice around prophylactic antibiotic use in lambs [61]. Farmers may not listen to their veterinarian’s advice because of their resistance to change their practices. They ignore feedback that disagrees with their chosen behaviour. As questions on quality of veterinary advice were not included in the survey, we cannot determine the quality of advice the farmers in this study received from their veterinarians. It is plausible that it may be the quality rather than the quantity of veterinary contact that may help to change farmers’ opinions on antibiotics. Further research is required to understand how we can change the behaviour of those that carry out traditional or habitual farming practices that are no longer recommended.

The median number of ewes was larger in the survey population than the national average [62] and may not be representative of the British sheep industry as a whole. However, the sample is likely to be representative of sheep farmers who supply lamb deadweight and who are aligned to a retailer. This was a general flock health survey that was not specifically based on antibiotic use. Responses therefore should not be biased towards those with a particular interest in antibiotic use. There may still be some selection bias as there may be differences in the farmers who participated in the study compared with those who did not participate in the study. As practices were self-reported by the farmers, there may be a risk of recall bias. To reduce this, recall was enhanced in this study by including pictures of different types of antibiotics to aid memory and including indication oriented and drug oriented questions [63]. Additionally, the survey encouraged respondents to refer back to their medicine books rather than relying on memory. Social desirability bias may occur where respondents give responses that they believe are more socially acceptable, rather than reporting their true beliefs or practices [64]. This might arise around antibiotic use, which could be considered a sensitive topic. However, given that many respondents in this study did not provide socially acceptable answers (e.g. over a fifth using antibiotics in all lambs for prevention of disease), this may not be the case. Finally, this study used a cross-sectional design, to investigate the influence of behaviours on attitudes. Therefore, this study only measures the associations between behaviours and attitudes and does not infer causality. A longitudinal intervention study is required to further strengthen the evidence that past behaviours can influence farmers’ attitudes towards antibiotics and understand whether there is a causal link.

### Implications for antibiotic behaviour change

One potential way of achieving recommended behaviours is through sharing positive attitudes towards antibiotic stewardship [65]. For those that are already using antibiotics appropriately, sharing positive attitudes towards antibiotic stewardship will increase and reinforce the consonance between their behaviour and attitudes and the behaviour will remain constant. For farmers who are using antibiotics for prevention of disease, an improved attitude towards antibiotic stewardship may increase the disagreement between the behaviour and attitudes [60]. This may lead to a reduction in prophylactic antibiotic use.

There are several initiatives to promote responsible antibiotic use. For example, the Antibiotic Guardian campaign is a worldwide information campaign to increase engagement and awareness of antibiotic resistance in healthcare professionals and the public, including farmers [66]. In the sheep sector the ‘Better Returns’ knowledge exchange publications published by AHDB provide information on responsible antibiotic use in lambs [39,67]. However, if behaviours are carried out due to habit, then information campaigns will not work as the information will not be received by those who it is aimed at [68,69]. Instead, interventions that change behaviour through disrupting the contextual cues to enact a behaviour could be used [70,71]. Alternative strategies used to prevent disease in sheep need to become the salient choice. This means alternatives should be convenient and available to the farmer compared with antibiotics, which should be less prominent and less readily available. To break contextual cue-behaviour associations it may be useful to plan ahead by preparing responses to anticipated antibiotic use cues [70]. This could be incorporated into the annual flock health plans that farmers are required to complete as part of farm assurance schemes [72].

Behaviours could also be changed by targeting farmers’ self-identity. We suggest that past behaviour may infer farmers’ self-identity as a traditional or good farmer [46]. When a recommended behaviour is identity-incongruent, then farmers may believe that behaviour is "not for people like me" and will not carry out the behaviour [73]. Thus, recommended practices need to be shown to link with identities of a productive, profitable farmer. Further research is required to understand the broad practices involved with shaping a farmers identity to inform identity-based interventions. Finally, many practices not directly related to antibiotic use were associated with farmers’ antibiotic attitudes. There may be a dynamic interdependency between farming practices [24], and it may be possible to change antibiotic use behaviours through targeting other behaviours such as lameness control, anthelmintic control or computer recording. This is called behavioural spillover [74]. Therefore, interventions may be measured by their effectiveness for multiple behaviours, rather than just a single direct behaviour.

### Conclusion

Overall, farmers who had antibiotic attitudes that were discordant with current antimicrobial use recommendations carried out more traditional practices, which was strengthened by their positive perceptions of their profitability. This may be explained by cognitive dissonance theory where farmers matched their attitudes to the behaviours they carried out. Additionally, habit may play an important role in farmers having attitudes that did not align with optimal antibiotic use recommendations; whereas veterinary contact did not influence farmers’ attitudes towards antibiotics. These results suggest that potential behaviour change interventions focussed on refining antibiotic use should challenge habitual behaviours through targeting farmers’ self-identity, disrupting the contextual cues of antibiotic use and improving positive attitudes towards antibiotic stewardship.
